# Synergistic Antioxidant and Anti-Inflammatory Effects between Modified Citrus Pectin and Honokiol

**DOI:** 10.1155/2017/8379843

**Published:** 2017-08-16

**Authors:** Cheppail Ramachandran, Barry Wilk, Steven J. Melnick, Isaac Eliaz

**Affiliations:** ^1^Dharma Biomedical LLC, 12777 Old Cutler Rd, Coral Gables, FL 33156, USA; ^2^Department of Pathology, Nicklaus Children's Hospital, Miami Children's Health System, 3100 SW 62nd Ave, Miami, FL 33155, USA; ^3^Econugenics, Inc., 396 Tesconi Court, Santa Rosa, CA 95401, USA; ^4^Amitabha Medical Clinic and Healing Center, 398 Tesconi Court, Santa Rosa, CA 95401, USA

## Abstract

Inflammation is a normal physiological process; however, dysregulation of this process may contribute to inflammatory-based chronic disorders and diseases in animals and humans. Therefore, the antioxidant and anti-inflammatory properties of natural products, often recognized in traditional medicine systems, represent therapeutic modalities to reduce or prevent uncontrolled inflammatory processes which in turn potentially ameliorate or prevent sequelae of inflammatory-based symptoms of chronic diseases. We have investigated the antioxidant and anti-inflammatory effects of honokiol (HNK) and modified citrus pectin (MCP)* in vitro* and examined whether the MCP : HNK combination has synergistic effects on antioxidant and anti-inflammatory properties. Although both HNK and MCP induced a dose-dependent increase in antioxidant activity, the latter has a consistently higher antioxidant effect. The MCP : HNK (9 : 1) combination induced a synergistic effect on antioxidant activity suggesting that the combination is significantly more efficacious than individual compounds. In mouse monocytes, the lipopolysaccharide- (LPS-) induced tumor necrosis-*α* (TNF-*α*) synthesis was significantly inhibited by HNK and the MCP : HNK combination in a dose-dependent manner and synergistic effects were clearly demonstrated with the combination on TNF-*α* inhibition. This combination effect was also evident on inhibition of nuclear factor-kappa B activity, cyclooxygenase-II activity, and lipid peroxidation in mouse monocytes. Further research into the combination is warranted.

## 1. Introduction

Dysregulated inflammation is often implicated as a pathophysiological phenomenon underlying many chronic diseases in humans and animals. Biomarkers associated with the responses are those that are often involved in the mediation of inflammation: proinflammatory cytokines, nitric oxide, and lipid mediators including cyclooxygenase enzymes and NF-*κ*B factors produced by inflammatory cells [1, 2]. Inflammation is in part characterized by the activation of the subsets of the innate immune system, such as monocytes and macrophages, and the secretion of inflammatory mediators like tumor necrosis factor-*α* (TNF-*α*), prostaglandin E2 (derived from cyclooxygenase-II), and nitric oxide [3]. Plant-derived natural products with antioxidant and anti-inflammatory properties are thus potentially beneficial for prevention and treatment of inflammation-associated chronic diseases.

Honokiol (HNK) is a biphenolic neolignan from magnolia bark* (Magnolia officinalis)* possessing multiple biological activities including antioxidant, anti-inflammatory anxiolytic, antidepressant, and neuroprotective properties [4–7]. It is widely used in Traditional Chinese Medicine and has already been shown in preclinical studies to be an effective multifunctional antioxidant, used for a wide variety of conditions including dermatological disorders [8], cancer prevention and therapeutics [4], neuromodulation [9], and cardiovascular conditions [10]. HNK at high concentrations is also reported to have anticancer and antiangiogenic properties whereas, at low doses (<10 *μ*M), it is a potent scavenger of super oxide and peroxyl radicals [11]. It contains two phenolic groups which can exhibit antioxidant properties like vitamin E. HNK inhibits freed radical-induced lipid peroxidation [12] and prevents oxidative modification of low-density lipoprotein (LDL), reducing the oxLDL-induced cytotoxicity, apoptotic features, and expression of adhesion molecules in endothelial cells [13].

Modified citrus pectin (MCP) is a well-investigated compound characterized from citrus peels that is recommended as a therapeutic agent for immune support [14], cancer [15–21], heavy metal toxicity [22–24], and fibrotic diseases [25–33]. The mechanism of action of MCP in inflammation, fibrosis, and cancer progression is that it works as a competitive inhibitor of extracellular galectin-3 (Gal-3). MCP blocks Gal-3 activity that drives fibrosis by reducing macrophage activity, proinflammatory cytokine expression, and apoptosis, as well as lowering inflammatory markers, thereby reducing tissue fibrosis. At elevated levels in the circulation, Gal-3 is involved in myofibroblast proliferation, inflammation and fibrogenesis, tissue repair, and ventricular and tissue remodeling [34, 35]. An elevated blood level of Gal-3 is associated with higher risk of death in acute decompensated heart failure and chronic heart failure patients [36] and also associated with the progression of autoimmune disease in studies of rheumatoid arthritis [37].

In the inflammatory cascade, the role of TNF-*α* and NF-*κ*B activity is well understood [1, 38]. The cytokine, TNF-*α*, mediates early-stage responses of inflammation by regulating the production of other cytokines, including interleukin-1 (IL-1) and IL-6. Because TNF-*α* is the main mediator of several inflammatory toxic responses to chemicals, it represents a promising target for the prevention of uncontrolled inflammation. TNF-*α* has also been reported to induce NF-*κ*B production and this protein is inhibited by the presence of antioxidants [39, 40]. Because the expression of many inflammation-associated genes, including iNOS, COX-2, and TNF-*α*, are known to be modulated by the binding of NF-*κ*B to its specific promoter regions, it represents a good target for suppressing NF-*κ*B activity for the regulation of lipopolysaccharide- (LPS-) induced inflammation [41]. In the present investigation, we have analyzed the anti-inflammatory and antioxidant properties of MCP, HNK, and the MCP : HNK combination to assess potential synergy for anti-inflammatory and antioxidant properties. A ratio of 9 parts of MCP with 1 part of HNK was used based on a profile of clinically relevant dosages, reduced side effects, and bioavailability data on MCP and HNK for a practical and effective oral capsule delivery use.

## 2. Materials and Methods

### 2.1. Test Compounds

Stock solution and dilutions of honokiol (HNK, HonoPure®, ecoNugenics, Santa Rosa, CA) and Modified Citrus Pectin (PectaSol-C®, ecoNugenics, Santa Rosa, CA) were prepared in sterile phosphate buffered saline (PBS) for treatment. The MCP : HNK combination was prepared in a ratio of 9 parts of MCP to 1 part of HNK (i.e., nine grams of MCP mixed with 1 gram of HNK) and dilutions of the mix were prepared in PBS. Test compounds were prepared at the highest concentration of 100 mg/ml in sterile PBS at 60°C. The amount of compounds that have been dissolved is calculated based on the weight of undissolved residue separated by centrifugation. The solubility of MCP and HNK in PBS was 76.4% and 100%, respectively. The volume was adjusted to get accurate amount of the compound for treatment based on solubility factor.

### 2.2. Antioxidant Activity

The principle of the antioxidant assay is based on the formation of a ferryl myoglobin radical from metmyoglobin and hydrogen peroxide, which oxidizes the 2,2′-azino-bis(3-ethylbenzthiazoline-6-sulfonic acid) (ABTS) to produce a radical cation, ABTS^+^, a soluble chromogen that is green in color and can be measured in a spectrophotometer at 405 nm. Antioxidants suppress the production of the radical cation in a concentration dependent manner and the color intensity decreases proportionally. Trolox, a water-soluble vitamin E analog, was used as a control antioxidant for analyzing the antioxidant activity of HNK, MCP, and MCP : HNK (9 : 1). In a 96-well plate, the assay was set up with 10 *µ*l of increasing concentrations of compounds (0–500 *µ*g/ml) and 20 *µ*l of myoglobin working solution per the protocol described in Antioxidant Assay Kit (Sigma-Aldrich, St. Louis, MO). Afterward, 150 *μ*l of ABTS working solution containing 0.0075% H_2_O_2_ was added and incubated at room temperature for 5 min. The reaction was stopped by adding 100 *µ*l stop solution and absorbance was measured at 405 nm in a Bio-Rad Benchtop microplate reader. The decrease in absorbance indicated the antioxidant activity of HNK, MCP, and MCP : HNK (9 : 1) equivalent to the Trolox standard, which was plotted against concentrations of compounds [42].

### 2.3. Lipopolysaccharide-Induced TNF-*α* Synthesis

Mouse monocytes (0.5 × 10^6^/ml) were plated in 24-well plates and starved overnight by growing in minimal essential medium containing 0.5% fetal bovine serum and antibiotics. On the following day, the plates were replaced with fresh starving medium and treated with increasing concentrations of HNK, MCP, and MCP : HNK (9 : 1) in the presence and absence of LPS. The compound was added initially and after incubation for 2 h at 37°C; 20 ng/ml LPS was added to induce an inflammatory response. The plate was incubated for an additional 4 h and culture medium was collected, centrifuged, and stored at −80°C. TNF-*α* produced and secreted into the medium by the cells was analyzed by ELISA protocol using the mouse TNF-*α* Quantikine ELISA kit (R&D systems, Minneapolis, MN) as per manufacturer's instructions [43].

### 2.4. Lipid Peroxidation

RAW 264.7 mouse monocytes (3 × 10^6^ cells/5 ml) were treated with increasing concentrations of MCP, HNK, and MCP : HNK (9 : 1) (0–2000 *µ*g/ml) at 37°C for 72 h before they were challenged with 20 *µ*M H_2_O_2_ overnight. The cell lysate was prepared per the Lipid Peroxidation Assay protocol (Sigma-Aldrich, St. Louis, MO) and protein concentrations were determined. The cell lysate (200 *µ*l) was analyzed for inhibition of lipid peroxidation per manufacturer's protocol. Lipid peroxidation is determined by the reaction of malondialdehyde (MDA) with thiobarbituric acid (TBA) to form a calorimetric (532 nm) product proportional to the MDA present. To form the MDA-TBA adduct 600 *µ*l of TBA solution was added to the 200 *µ*l of lysate and incubated at 95°C for 1 h. The reaction mix was cooled in an ice bath for 10 min and absorbance recorded at 532 nm in a Beckman spectrophotometer. MDA standards (0–2 nmoles) reacted with TBA were used as a standard for the calculation of lipid peroxidation activity. The reaction mix attains pink color and decrease in absorbance indicated the inhibition of adduct formation. The percentage of inhibition was calculated based on untreated cells and plotted against concentrations of compounds [42].

### 2.5. Nitric Oxide Synthesis

RAW 264.7 monocytes (1 × 10^6^ cells/ml) were seeded in starving phenol-free minimum essential medium (MEM) containing 0.5% FBS and antibiotics overnight in 24-well plates. On the following day, the starving medium was replaced with fresh medium and cells were treated with increasing concentrations of compounds for 2 h followed by NO stimulation with LPS (20 ng/ml) for a total of 24 h. Supernatants were collected after centrifugation and used for analysis of nitrite and nitrate levels using the nitric oxide quantitation kit (Active Motif, Carlsbad, CA) [43].

### 2.6. NF-*κ*B Activation

RAW 264.7 cells (3 × 10^6^/5 ml) were incubated with increasing concentrations of HNK, MCP, and MCP : HNK (9 : 1) for 48 h and nuclear proteins were extracted with TransAM NF-*κ*B p65 activity ELISA kit (Active Motif, Carlsbad, CA) per manufacturer's instructions. Protein concentration of the nuclear lysate was determined and lysate equivalent to 20 *µ*g protein was analyzed for NF-*κ*B activity using the TransAM NF-*κ*B p65 activity kit. In the TransAM kit, the NF-*κ*B consensus site (5′-GGGACTTTCC-3′) is immobilized on the ELISA plate and the active form of NF-*κ*B contained in the nuclear extract will specifically bind to the nucleotide. The complex can be detected with NF-*κ*B primary and secondary antibody reactions followed by substrate color reactions. The ELISA plates were read at 450 nm with a reference wavelength of 655 nm in a microplate reader. The decrease in the absorbance compared to untreated sample indicated the inhibition of NF-*κ*B activity (%) which was plotted against concentrations of compounds [44].

### 2.7. Cyclooxygenase-II Activity

Cyclooxygenase (COX) activity assay kit from Abcam (Cambridge, MA) and COX-II enzyme from Sigma-Aldrich (St. Louis, MO) were used for analyzing the effect of MCH, HNK, and MCH:HNK (9 : 1) on COX-II activity. The assay kit uses a chemiluminescent substrate to detect the peroxidative activity of the COX-II enzyme. Ibuprofen (nonsteroidal anti-inflammatory drug, NSAID) is used as a positive control in the assay. After inhibition with increasing concentrations of MCP, HNK, or MCP : HNK (9 : 1), the residual activity of COX-II is measured by addition of a luminescent substrate and arachidonic acid. Light emission will start immediately and is directly proportional to the COX-II activity in the sample which is measured quickly by using a Veritas Luminometer (Turner Biosystems, Sunnyvale, CA) equipped with injectors for both substrate and arachidonic acid. The relative light units (RLU) recorded by the luminometer were used to calculate the percent inhibition of COX-II activity by MCH, HNK, or MCH:HNK (9 : 1) according to the following formula: percent inhibition (1 − average net inhibitor RLU/average net RLU for uninhibited) × 100. The inhibition percentage was plotted against PMF concentrations of compounds.

### 2.8. CompuSyn Analysis

To determine the synergistic/additive/antagonistic effects between MCP and HNK, data on various biochemical parameters were analyzed further using CompuSyn software (CompuSyn Inc., Paramus, NJ). This program is based on Chou and Talalay's multiple drug effect equations [45] and it defines synergism as a more-than-expected additive effect and antagonism as a less-than-expected additive effect. The combination index (CI) was calculated by the Chou-Talalay equations for multiple drug effects, which consider both potency (inhibitory concentration values) and shape (slope, *m*) of the dose-effect curve [45, 46].

### 2.9. Statistical Analysis

Mean and standard deviation estimates were calculated using Excel software. The data were analyzed statistically by paired *t* test (GraphPad Prism software, La Jolla, CA) and *p* values were used to determine the significant difference between treatment groups.

## 3. Results

### 3.1. Antioxidant Activity

**Figure 1:**
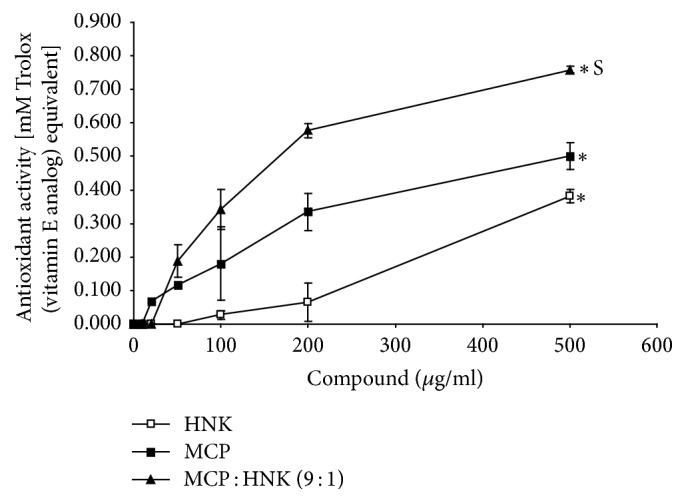
Antioxidant activity of HNK, MCP, and MCP : HNK (9 : 1). The activity equivalent to mM Trolox (vitamin E analog) was analyzed using Antioxidant Assay Kit (Sigma-Aldrich, MO) and plotted against compound concentrations. Antioxidant activity curves were statistically analyzed by paired *t* test; ^*∗*^*p* < 0.05 for HNK versus MCP, HNK versus MCP : HNK (9 : 1), and MCP versus MCP : HNK (9 : 1); S, synergism between MCP and HNK.

The results of antioxidant assay presented in Figure 1 showed that both HNK and MCP induced a dose-dependent increase in the antioxidant activity, the latter showing a consistently higher response than the former. MCP : HNK (9 : 1) mix showed significantly higher antioxidant activity than the single agents alone, especially at 200 and 500 *μ*g/ml concentrations.

### 3.2. Inhibition of LPS-Induced TNF-*α* Production

**Figure 2:**
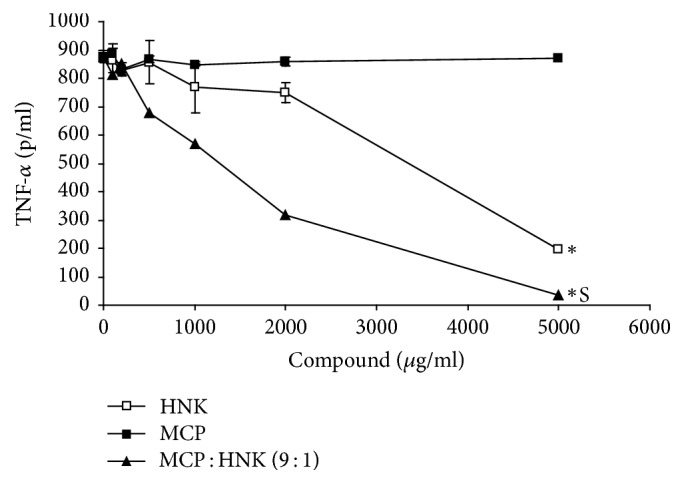
Inhibition of LPS-induced TNF-*α* (pg/ml) production by HNK, MCP, and MCP : HNK (9 : 1) in RAW 264.7 mouse monocyte cell line. The cells were treated with compounds and/or LPS in starvation medium and TNF-*α* analyzed by ELISA. Inhibition curves were analyzed by paired *t* test; ^*∗*^*p* < 0.05 for HNK versus MCP, and HNK versus MCP : HNK (9 : 1); S, synergism between MCP and HNK.

When RAW 264.7 mouse monocyte cells were treated with LPS (20 ng/ml), proinflammatory cytokine TNF-*α* is induced (875.35 pg/ml) and secreted into the medium. Treatment of mouse monocytes with increasing concentrations of HNK inhibited the LPS-induced TNF-*α* synthesis significantly in a dose-dependent manner (Figure 2). As compared to HNK, MCP showed essentially no inhibitory effect on LPS-induced TNF-*α* synthesis by monocytes. However, the MCP : HNK (9 : 1) combination showed significantly better inhibitory effect than HNK alone. The highest dose of MCP : HNK (9 : 1) (5000 ug/ml) has almost completely inhibited the LPS-induced TNF-*α* synthesis in monocytes.

### 3.3. Inhibition of Lipid Peroxidation

**Figure 3:**
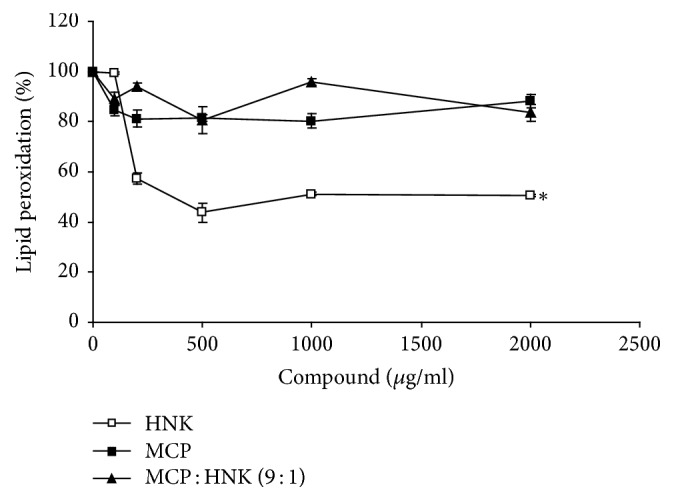
Inhibition of lipid peroxidation by HNK, MCP, and MCP : HNK (9 : 1) in RAW 264.7 mouse monocyte cell line. Lipid peroxidation was significantly inhibited by HNK treatment of monocytes. The treatment groups were compared using paired *t* test; ^*∗*^*p* < 0.05 for HNK versus MCP and HNK versus MCP : HNK (9 : 1). MCP and MCP : HNK (9 : 1) treatments were not statistically significant.

The effect of HNK, MCP, and MCP : HNK (9 : 1) on H_2_O_2_-induced lipid peroxidation is shown in Figure 3. HNK has significant inhibitory effect on lipid peroxidation with 58% inhibition at 500 *μ*g/ml concentration. Higher concentrations of HNK beyond 500 *μ*g/ml dose do not inhibit the lipid peroxidation further. MCP and MCP : HNK (9 : 1) mix inhibit lipid peroxidation slightly with 20% reduction at 500 *μ*g/ml dose. MCP demonstrated a steady inhibition of lipid peroxidation up to 200 *μ*g/ml dose which remained stable with increase in dosage.

### 3.4. Inhibition of NF-*κ*B Activity

**Figure 4:**
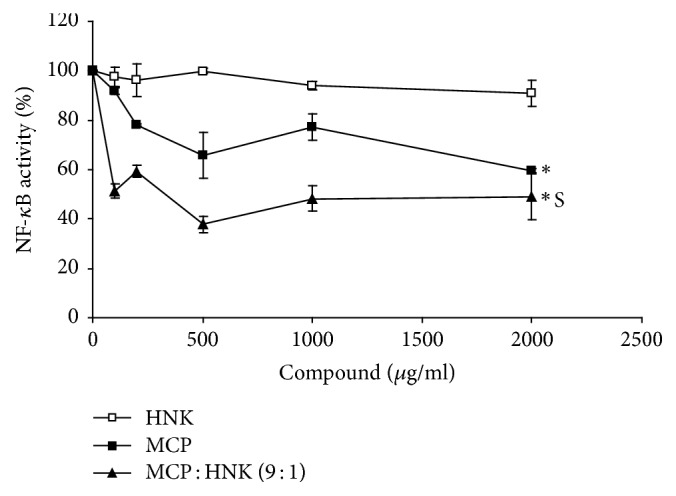
Inhibition of NF-*κ*B (p65) activity by HNK, MCP, and MCP : HNK (9 : 1) in RAW 264.7 mouse monocyte cell line. NF-kB activity is inhibited significantly by MCP and MCP : HNK (9 : 1) treatment of monocytes and not by HNK alone. The treatment groups were compared using paired *t* test; ^*∗*^*p* < 0.05 for HNK versus MCP and HNK versus MCP : HNK (9 : 1). No significant difference was observed between MCP and MCP : HNK (9 : 1) treatment. S, synergism between MCP and HNK.

The results of NF-*κ*B activity assay presented in Figure 4 showed that HNK treatment of RAW 264.7 mouse monocytes has no significant inhibitory effect on NF-*κ*B activity. However, MCP inhibited the NF-*κ*B activity significantly with about 35% and 40% inhibition at 500 ug/ml and 2000 *μ*g/ml doses, respectively. On the other hand, MCP : HNK (9 : 1) combination demonstrated the highest inhibitory effect at every concentration, with about 60% inhibition at 500 ug/ml and 2000 *μ*g/ml doses.

### 3.5. Inhibition of COX-II Activity

**Figure 5:**
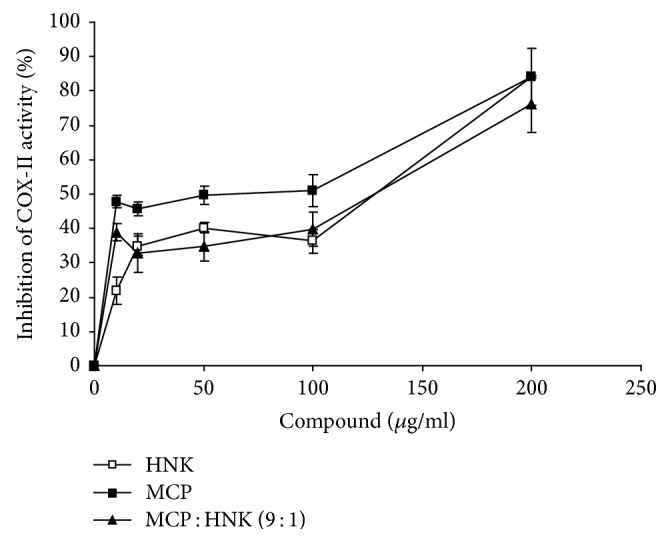
Inhibition of cyclooxygenase-II (COX-II) activity by HNK, MCP, and MCP : HNK (9 : 1) treatment. The inhibition curves were compared by paired *t* test; *p* < 0.05 for HNK versus MCP and MCP versus MCP : HNK (9 : 1). No significant difference was observed between MCP and MCP : HNK (9 : 1) treatment.

HNK, MCP, and MCP : HNK (9 : 1) inhibit COX-II activity (Figure 5). The inhibition level plateaued between 10 and 100 *μ*g/ml and then further increased at a concentration >200 *μ*g/ml in all treatment groups. MCP alone demonstrates greater inhibition profile than HNK and MCP : HNK (9 : 1) combination, with about 85% inhibition of COX-II activity at 200 *μ*g/ml concentration.

### 3.6. Effect of HNK, MCP, and MCP : HNK (9 : 1) on Nitric Oxide Synthesis

**Figure 6:**
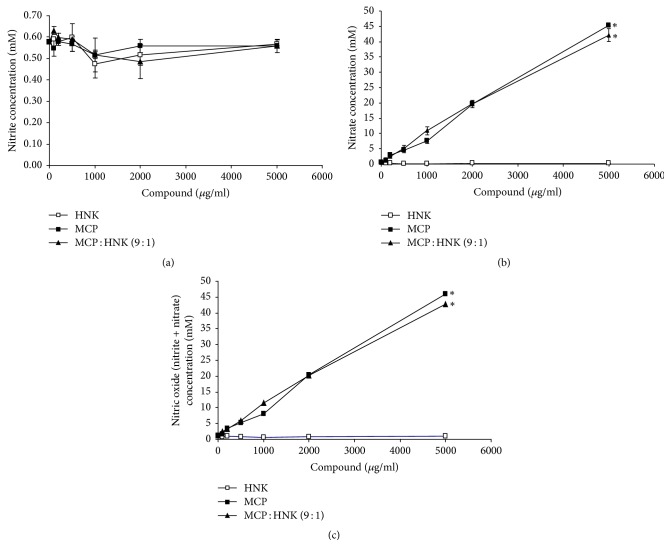
Effect of HNK, MCP, and MCP : HNK (9 : 1) on nitric oxide synthesis in RAW 264.7 mouse monocyte cell line. (a) Effect on nitrite concentration in cells treated with increasing concentrations of compounds. No significant difference was noticed among HNK, MCP, and MCP : HNK (9 : 1) treated monocytes. (b) Effect on nitrate concentration in cells treated with increasing concentrations of HNK, MCP, and MCP : HNK (9 : 1). Treatment groups were compared using paired *t* test; ^*∗*^*p* < 0.05 for HNK versus MCP and HNK versus MCP : HNK (9 : 1). No significant difference between MCP and MCP : HNK (9 : 1) treatment groups. (c) Effect on nitrite + nitrate concentration in cells treated with increasing concentrations of HNK, MCP, and MCP : HNK (9 : 1). Treatment groups were compared using paired *t* test; ^*∗*^*p* < 0.05 for HNK versus MCP and HNK versus MCP : HNK (9 : 1). No significant difference is noticed between MCP and MCP : HNK (9 : 1).

The concentration of nitrite and nitrate levels is an indication of nitric oxide synthesis. The effect of HNK, MCP, and MCP : HNK (9 : 1) on nitrite and nitrate concentration in RAW264.7 mouse monocytes is presented in Figures 6(a)–6(c). All three compounds failed to show any significant effect on nitrite synthesis. However, MCP and the MCP : HNK (9 : 1) mixture elevated the nitrate concentration in a dose-dependent manner, without any significant difference between the groups. In the case of HNK treated cells, nitrate level remained the same without any change and the data matched nitrite concentration. It is quite possible that nitrogen content in MCP may be contributing to the spike in nitrate levels.

### 3.7. Synergism between MCP and HNK

Table 1 shows the dose-effect relationship between MCP and HNK for various biochemical parameters analyzed in the present investigation. The combination index (CI) estimates demonstrated synergism between MCP and HNK for inhibition of LPS-induced TNF-*α* because the CI values were below 1 at ED_50_, ED_75_, and ED_90_ levels. The drug reduction index (DRI) indicated the potential reduction of MCP and HNK amounts to achieve an ED_50_ effect. A synergistic effect between MCP and HNK was also observed for antioxidant activity with CI values between 0.3 and 0.7 (synergism) at ED_50_, ED_75_, and ED_90_ levels. The DRI values for antioxidant effect indicated the dose reduction possible by combining MCP and HNK. For lipid peroxidation, MCP and HNK combination has additive effect at an ED_50_ level and low level of antagonism at ED_75_ and ED_90_ levels. CompuSyn analysis also showed strong level of synergism between MCP and HNK for inhibition of NF-*κ*B activity.

## 4. Discussion

Overproduction of inflammatory mediators such as NF-*κ*B, TNF-*α*, COX-II, and NO produced by macrophages, neutrophils, and other immune cells is very much involved in the pathogenesis of chronic diseases, for example, atherosclerosis, arthritis, type 2 diabetes, and cancer [47–49]. Therefore, identification of natural product-derived extracts or compounds that controls the production of inflammatory mediators is an extremely attractive therapeutic or preventative modality for these diseases [50]. In the present investigation, both HNK and MCP treatment have enhanced the antioxidant activity in monocytes. Furthermore, by using CompuSyn analysis we showed that the combination of MCP and HNK has synergistic effect on the antioxidant activity of monocytes. The CI values of the MCP : HNK (9 : 1) combination for antioxidant activity are <1 at ED_50_, ED_75_, or ED_90_ levels, thereby indicating the strong synergism between MCP and HNK for antioxidant activity. Such synergistic effects for antioxidant activity have been observed for combinations of extracts from spices (anise, cardamom, clove, and cinnamon) [51] and also between black tea and curcumin [52], which may form the basis for a therapeutic that has superior effects compared to single agents.

The dysregulation of the redox system is implicated in numerous human disorders including neoplasia, inflammation, degenerative diseases, and environmental exposures [53, 54]. HNK has been shown to reduce intracellular super oxide 2-fold and therefore free radical scavenging activity is implicated as an important attribute of HNK [11]. Further, HNK inhibits copper-induced oxidative modification of LDL [12] and iron-stimulated lipid peroxidation [13]. It is also known that oxygen overproduction stimulates lipid peroxidation and the present investigation showed that HNK inhibits lipid peroxidation which may involve both initiation and propagation of peroxidation process [12].

NF-*κ*B regulates the transcription of several genes, including iNOS, COX-II, TNF-*α*, and IL-6 and is thus important for the development of inflammation-associated diseases [1, 50, 55]. NF-*κ*B is primarily composed of proteins with molecular mass of 50 (p50) and 65 kDa (p65) and is retained in the cytoplasm by inhibitor of *κ*B (I-*κ*B). From its unstimulated form, NF-*κ*B is activated by a wide variety of inflammatory stimuli like LPS. Most of these inflammatory mediators induce the phosphorylation-dependent degradation of I-*κ*B proteins, allowing active NF-*κ*B to translocate into the nucleus, where it regulates several genes including iNOS, COX-II, TNF-*α*, and IL-6 [55]. In the present investigation, we have shown that both MCP and MCP : HNK (9 : 1) combination have significant inhibitory effect on NF-*κ*B activity whereas HNK alone showed little inhibition of NF-*κ*B activity. Furthermore, very strong synergism between MCP and HNK for inhibition of NF-*κ*B activity and also the DRI values (Table 1) may facilitate the reduction of MCP and HNK amounts in the combination to achieve the defined therapeutic effect. The inhibition profile of TNF-*α* by these compounds also follows a similar pattern as NF-*κ*B activity. HNK demonstrated the strongest inhibitory effect whereas MCP failed to show any inhibition of TNF-*α* unlike NF-*κ*B. However, CompuSyn analysis showed the synergism between MCP and HNK for TNF-*α* -inhibition with CI values of <1 at ED_50_, ED_75_, and ED_90_ levels which were similar to NF-*κ*B inhibition. Furthermore, all three compounds including the MCP : HNK (9 : 1) combination inhibited COX-II activity. The MCP : HNK combination does show an additive effect for inhibition of lipid peroxidation at ED_50_ level. In summary, MCP : HNK combination appears to be superior to individual agents due to the synergism for enhanced antioxidant activity, inhibition of NF-*κ*B activity, and LPS-induced TNF-*α* synthesis.

## 5. Conclusion

The current investigation demonstrated the antioxidant and anti-inflammatory effects of MCP and HNK and the MCP : HNK in vitro. The MCP : HNK combination in general demonstrated significantly better effects than the single compounds due to the synergistic effects on antioxidant activity, inhibition of LPS-induced TNF-alpha production, and NF-*κ*B activity. This demonstration of synergistic antioxidant and anti-inflammatory properties of MCP and HNK suggests a potential application for the amelioration of effects due to oxidative stress and free radical damage.

## Figures and Tables

**Table 1:** Dose-effect relationship between modified citrus pectin (MCP) and honokiol (HNK).

Biochemical traits	CI at ED_50_	CI at ED_75_	CI at ED_90_	DRI-MCP ED_50_	DRI-HNK ED_50_
Inhibition of LPS-induced TNF-*α*	0.549	0.515	0.510	1.825	706.268
Antioxidant activity	0.670	0.546	0.446	33.682	1.562
Inhibition of lipid peroxidation	1.070	1.765	1.189	—	—
Nitric oxide synthesis (NO_2_ + NO_3_)	2.932	4.675	5.684	—	—
Inhibition of NF*κ*B activity	0.068	0.131	0.254	14.749	917.283
Inhibition of COX-II activity	2.248	3.080	1.870	—	—

CI, combination index; DRI, drug reduction index; ED, effective dose; CI values by CompuSyn analysis: <0.1: very strong synergism; 0.1–0.3: strong synergism; 0.3–0.7: synergism; 0.7–0.85: moderate synergism; 0.85–0.90: slight synergism; 0.90–1.10: nearly additive; 1.10–1.20: slight antagonism; 1.20–1.45: moderate antagonism; 1.45–3.3: antagonism; 3.3–10: strong antagonism; >10: very strong antagonism.

## Abbreviations

ABTS: 2,2′-azino-bis(3-ethylbenzthiazoline-6-sulfonic acid)
CI: Combination index
COX-II: Cyclooxygenase-II
DRI: Drug reduction index
ELISA: Enzyme linked immunosorbent assay
ED: Effective dose
Gal-3: Galectin-3
H_2_O_2_: Hydrogen peroxide
HNK: Honokiol
I-*κ*B: Inhibitor of *κ*B
IL-1: Interleukin-1
IL-6: Interleukin-6
iNOS: Inducible nitric oxide synthase
LDL: Low-density lipoprotein
LPS: Lipopolysaccharide
MCP: Modified citrus pectin
MDA: Malondialdehyde
MEM: Minimum essential medium
NF-*κ*B: Nuclear factor-kappa B
NSAID: Nonsteroidal anti-inflammatory drug
PBS: Phosphate buffered saline
TBA: Thiobarbituric acid.
